# Household Food Insecurity and Parenting Confidence and Stress among Women in the Democratic Republic of Congo

**DOI:** 10.51894/001c.163953

**Published:** 2026-08-01

**Authors:** Laasya Koduri, Noor Msallaty, Michael Boivin, Desire Tshala, Itziar Familiar-Lopez

**Affiliations:** 1 College of Osteopathic Medicine, Michigan State University

## 01

### BACKGROUND

Household food insecurity remains a major global public health challenge and has been linked to increased psychological distress. Less is known about how food insecurity affects parenting stress and sense of confidence in low- and middle-income countries.

### OBJECTIVES

To examine the relationship between household food insecurity and parenting stress, parenting confidence, and mental health symptoms among women in the Democratic Republic of Congo (DRC).

### METHODS

Secondary analysis of baseline data from a longitudinal study examining mental health outcomes among women in Kahemba, DRC. Household food insecurity was measured using the Household Food Insecurity Access Scale (HFIAS). Parenting stress was measured using the Parental Stress Scale (PSS), and parenting confidence was assessed using the Parent Sense of Competence Scale (PSOC). Mental health symptoms were assessed using the DSM-5-TR Self-Rated Level 1 Cross-Cutting Symptom Measure, classifying participants as DSM-positive if they endorsed symptoms meeting the threshold for further clinical attention in at least one domain. Descriptive statistics were calculated with t-tests to evaluate differences in means, and bivariate linear regressions were used to examine HFIAS score as a predictor of PSS, PSOC, and logistic regression for DSM-positive symptoms, separately.

### RESULTS

The sample included 227 women with a mean age of 39.2 years and mostly married (68%). Mean HFIAS score was 7.5 (moderate food insecurity) and was higher among single than married women (14.07 vs. 7.42, *p*<0.0001). Women had low PSOC scores (average=56.8) and low PSS (38.7). Eighty-seven percent of women (164) could be classified as having any DSM-positive domain, and the most frequent domains reported were substance use (39.9%), somatic symptoms (64.4%), and anxiety (58.5%). Greater household food insecurity was associated with higher levels of parenting stress (β=1.09, *p*<0.001) and higher levels of parenting confidence (β=1.16, *p*<0.001). Women classified as having any DSM-positive symptoms had higher food insecurity scores, compared to those who did not (β=0.14, *p*=0.001).

### DISCUSSION

Findings suggest that food insecurity may play an important role in shaping parenting stress and confidence as well as maternal mental health, underscoring the need for integrated research and interventions that consider household economic stressors when addressing caregiver mental health and parenting outcomes.

